# On the onset and dispersal of a major MDR TB clone among HIV-negative patients, Tunisia

**DOI:** 10.1186/s13756-023-01360-7

**Published:** 2024-02-14

**Authors:** Naira Dekhil, Helmi Mardassi

**Affiliations:** Unit of Typing and Genetics of Mycobacteria, Laboratory of Molecular Microbiology, Vaccinology, and Biotechnology Development, Pasteur Institute, Tunis, University of Tunis El Manar, Tunis, Tunisia

**Keywords:** *Mycobacterium tuberculosis*, MDR/XDR TB, Outbreak, XDR phenotype, Incarceration, Whole genome sequencing, Phylogeography, Dating analysis

## Abstract

**Background:**

To carry out a whole genome sequencing (WGS)-based investigation on the emergence and spread of the largest multidrug-resistant tuberculosis (MDR TB) outbreak that has been thriving among HIV-negative patients, Tunisia, since the early 2000s.

**Methods:**

We performed phylogeographic analyses and molecular dating based on a WGS dataset representing 68 unique *Mycobacterium tuberculosis* isolates, covering almost the entire MDR TB outbreak for the time period 2001–2016.

**Results:**

The data indicate that the ancestor of the MDR TB outbreak emerged in the region of Bizerte, as early as 1974 (95% CI 1951–1985), from where it spread to other regions by 1992 (95% CI 1980–1996). Analysis of a minimum spanning tree based on core genome Multilocus Sequence Typing (cgMLST) uncovered the early spill-over of the fitness-compensated MDR TB strain from the prison into the general population. Indeed, cases with history of incarceration were found to be directly or indirectly linked to up to 22 new outbreak cases (32.35%) among the non-imprisoned population. By around 2008, the MDR TB outbreak strain had acquired additional resistance, leading to an XDR phenotype.

**Conclusions:**

WGS allowed refining our understanding of the emergence and evolution of the largest MDR TB outbreak in Tunisia, whose causative strain has been circulating silently for almost 26 years before. Our study lends further support to the critical role of prisons-related cases in the early spread of the outbreak among the general population. The shift to an XDR phenotype of such an epidemic clone prompts an urgent need to undertake drastic control measures.

**Supplementary Information:**

The online version contains supplementary material available at 10.1186/s13756-023-01360-7.

## Background

Controlling the expansion of drug-resistant *Mycobacterium tuberculosis* represents a global challenge of upmost importance to health security. Of major concern is multidrug-resistant tuberculosis (MDR TB), which is defined as TB that is resistant to at least isoniazid and rifampicin, the two most powerful first-line TB drugs. With an estimated global success rate of 59% [1], not only is MDR TB difficult to treat, but it could also evolve into an even worse form, extensively drug-resistant TB (XDR TB), by accumulating additional resistance to any fluoroquinolone and to at least one additional Group A drug (levofloxacin, moxifloxacin, bedaquiline and linezolid) [2].

Because XDR TB displays resistance to the most potent TB drugs, the remaining treatment alternatives are less effective, cause more toxicity, and incurs exorbitant costs [3]. Furthermore, the global fight against MDR and XDR TB is hampered by deficiencies in health services, particularly in low and middle income countries, the ascending rate of HIV co-infection, and the recent impact of the COVID-19 pandemic [4].

In Tunisia, the proportion of incident MDR TB cases is rather low (1% in 2021) [1], being mostly dominated by cases related to a major MDR TB Outbreak, which has been identified in the region of Bizerte in the early 2000s. This outbreak is caused by a sublineage L4.1.2.1/Haarlem genotype strain, which by 2011, involved 48 individuals (mean age 31.09 yrs; 89.6% male) [5, 6]. A hallmark of this MDR TB outbreak is the fact that it has emerged and thrived, from the outset, in a context strictly negative for HIV infection, suggesting its intrinsic increased fitness and/or virulence. Strikingly, the involved MDR TB outbreak strain was found to harbour a mutation within *rpoB*, *rpoB* V615M, secondary to the rifampicin resistance-conferring mutation *rpoB* S531L, which has been shown to compensate for the incurred fitness cost, while conferring high-level resistance to rifampicin. This double *rpoB* mutation was thus proposed to be a major determinant contributing to the epidemic potential of this MDR TB outbreak strain [7].

The Bizerte’s Haarlem MDR TB outbreak was characterized by high rates of fatality and relapse as demonstrated after following patients for more than 11 years. Indeed, 70% of those whose treatment failed have died, and 24% among those deemed successfully treated have relapsed [5]. More worryingly, the outbreak strain has evolved into an XDR phenotype by 2010 and has been transmitted thereafter to other patients, which could alert on the emergence of highly transmissible XDR TB strains in this region [5, 8].

Whole-genome sequencing (WGS) is now becoming the gold-standard for typing *M. tuberculosis* isolates given its higher resolution over MIRU-VNTR-based clustering. The use of WGS in MDR TB outbreak investigations generates important insights into the transmission dynamics, providing predictions of the extent and direction of transmission, as well as the outbreak’s evolutionary trend [9, 10]. With regards to drug resistance, WGS has significantly improved our understanding of causal mechanisms and the compensatory process that mitigate the deleterious effects of drug resistance on the reproductive capacity and/or transmission (fitness) of the bacillus [11].

In this study, we performed a WGS-based study spanning a 16-year period aimed at refining our understanding of the circumstances surrounding the onset and spread of this major Haarlem MDR TB outbreak.

## Methods

### WGS dataset

The study sample consists of 68 WGS data representative of eight regions in Tunisia, including 39 newly sequenced genomes of MDR TB outbreak isolates collected from different HIV-negative patients between years 2001 to 2011 (Additional file 1: Table S1). These 39 newly sequenced genomes represent the very first dispersal events of the MDR TB outbreak since its identification in year 2001. The corresponding isolates were cultured from sputum samples that were forwarded to the Unit of Typing & Genetics of Mycobacteria of the Institut Pasteur, Tunis, as part of its routine diagnostic activity. Isolation, drug susceptibility testing (DST) and genotyping of these isolates have previously been described by our laboratory in Dekhil et al. (2016). The remaining 33 WGS data are publicly available and correspond to isolates of the same MDR TB outbreak that were recovered from different patients during the time period 2012 to 2016 [8] (Additional file 2: Table S2).

### Whole genome sequencing

Genomic DNA from cultured bacteria was isolated by phenol/chloroform extraction [12] and subjected to WGS. Genomic Libraries and sequencing were performed by Biomics, Institut Pasteur Paris (https://research.pasteur.fr/fr/team/biomics/) using Illumina Nextseq 500[13]. Raw data of sequenced isolates was submitted to the European Nucleotide Archive under project number PRJEB63074.

Illumina paired-end sequencing data were analysed using a pipeline composed of open source software as described previously [14]. Briefly, raw sequencing data were checked for quality control using FASTQC [15]. Trimming of adapters and low-quality bases with a Phred quality score of less 20 and filtering for a minimum read length of 36 were done using Trimmomatic [16]. Paired-End reads were mapped to the genome sequence of *M. tuberculosis* H37Rv reference strain (Genbank: AL123456) using three different alignment algorithms namely the Burrows-Wheeler Alignment Tool (BWA) [17], Novoalign [18] and SMALT [19]. Local realignment and de-duplication of alignment files were done using the Genome Analysis Toolkit (GATK) [20] and Picard [21]. Single nucleotide polymorphisms (SNPs) and Insertion/Deletions (In/Dels)) were then identified from each alignment file using GATK. Variants were annotated using snpToolkit (https://github.com/Amine-Namouchi/snpToolkit). SNPs in PE/PGRS genes, mobile elements or repeat regions were excluded from our analysis.

### Phylogenetic analysis

To comply with data standardization recommended in epidemiological investigations, we used a core genome Multilocus Sequence Typing (cgMLST) scheme for clinical MTBC isolates based on 3,257 core genome genes (see Table S3 in the Additional file 3) [22]. SNPs were concatenated resulting in one character string (nucleotide sequence) and a FASTA file was created for each clinical isolate. Minimum Spanning Trees (MST) based on cgMLST was computed on the basis of an alignment of 690 polymorphic sites with GrapeTree software (https://achtmanlab.github.io/GrapeTree/MSTree_holder.html). Using an upper threshold of five SNPs between genomes, we identify recently linked isolates [23].

### Pairwise SNP distances

Pairwise SNP distance matrix from a FASTA sequence alignment concatenating all polymorphic sites (*N* = 969) was generated with snp-dist (https://github.com/tseemann/snp-dists), then plotted using R version 4.0.3. Wilcoxon rank sum test was used to test for differences between clones SNPs distance, as data did not show a normal distribution.

### Molecular dating

Divergence times and evolutionary rates were calculated using a molecular clock method based on least-squares optimisation, LSD2 (Least Squares Dating) [24]. The advantage of this method consists in its capacity to perform faster than fully Bayesian approaches. However to overcome the limitation of using a single tree topology and a strict clock, we generated a maximum likelihood tree based on 13,158 polymorphic sites, using Raxml [25] with 1000 bootstraps and selecting the best-scoring. Moreover, we performed a date randomization test with 100 randomized datasets using the quadratic programming dating (QPD) algorithm and calculated the confidence interval (options -f 100 and -s). Since the alignments contained only variable positions, we rescaled the branch lengths of the tree using Gotree version 0.4.4 (https://github.com/evolbioinfo/gotree). The phylogeny was rooted using *M. canetti*. The tree was annotated using iTOL version 5 [26].

## Results

### Drug resistance profiles

Sequencing data revealed a wide range of gene mutations associated with resistance to first-and second-line antituberculosis drugs. All patients had the same fixed INH, RIF, and SM resistant related gene mutations as previously described [5]. However, four clinical isolates (5.88%) evolved into an XDR phenotype, as they accumulated the ofloxacin resistance mutation *gyr*A A90V and the kanamycin/amikacine resistance mutation *rrs* A1401G. Four isolates showed mutations in the *fabG1* gene promoter region (15C > T or 8 T > C), conferring resistance to Ethionamide [27] (Table 1).

**Table 1:** Overview of phenotypic resistance of MDR strains to first-line and second-line antituberculosis drugs

Resistance	Number of strains (%)	Gene	Mutations conferring drug resistance
MDR	64 (94,18)		
XDR	4 (5,88)		
+ RIF	68 (100)	*rpoB*	S531L, V615M
+ EMB	68 (100)	*embB*	M306I
+ PZA	43 (63.23)	*pncA*	A-11C Q10H C42A G10P L4W *Indel_Gene_Pos 341 CGT/C *Indel_Gene_Pos 297 G/GC *Indel_Gene_Pos 29 CTGCACGTCGACG/C *Indel_Gene_Pos 342 A/AC
+ STM	68 (100)	*gidB*	R47W
+ INH	68 (100)	katG	S315T
+ ETO	4 (5.88)	*fabG1*	T-8C, C-15 T
+ AMG	4 (5.88)	*rrs*	A1401G
+ FQ	4 (5.88)	*gyrB*	D461H, G470C
+ PAS	0		
+ CS	0		

*EMB* Ethambutol, *PZA* Pyrazinamide, *STM* Streptomycin, *AMG* Aminoglycosides (kanamycin, amikacin, capreomycin), *ETO* Ethionamide, *FQ* Fluoroquinolones, *PAS* Para aminosalicylic acid, *CS* Cycloserine, Indel_Gene_Pos Insertion-Deletion_Gene_Position

### Pinpointing recent transmission chains

**Fig. 1:**
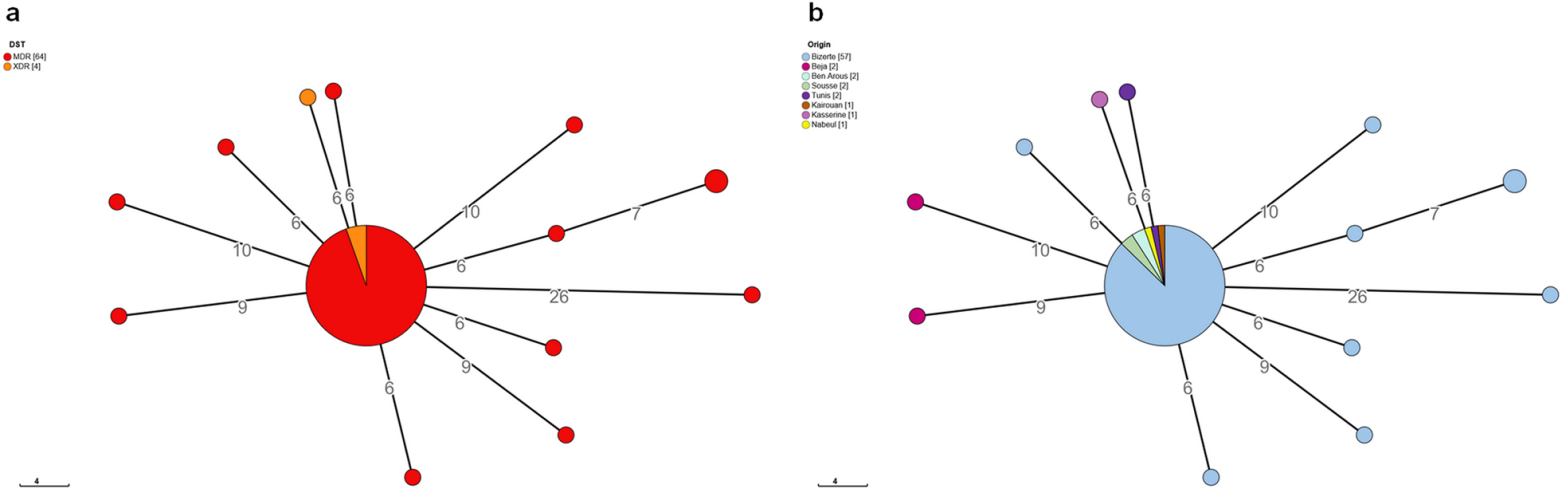
Core genome Minimum spanning tree (cgMLST) based on the concatenated sequences of the 690 polymorphic sites determined by WGS analysis. A cut-off of 5 SNPs has been used for the clustering of recently linked cases. Different colours are assigned to clusters according to DST **a** or Origin **b** Numbers correspond to SNP differences between two nodes in the tree

We sought to use the Minimum Spanning Tree (MST) based on concatenated cgMLST sequences of 68 genomes to identify clusters that reflect recent transmission. We used a 5 SNPs cgMLST allele clustering cut-off distance between isolates to detect recent transmission chains [23]. A large cluster of 55 isolates (52 MDR and 3 XDR isolates) was identified, yielding a clustering rate of 79.41%. This cluster included isolates from six geographic regions with the majority (85.45%) originating from the region of Bizerte. Likewise, the 13 outliers belonged mainly to Bizerte (Fig. 1B).

### Genomic epidemiological analysis

**Fig. 2:**
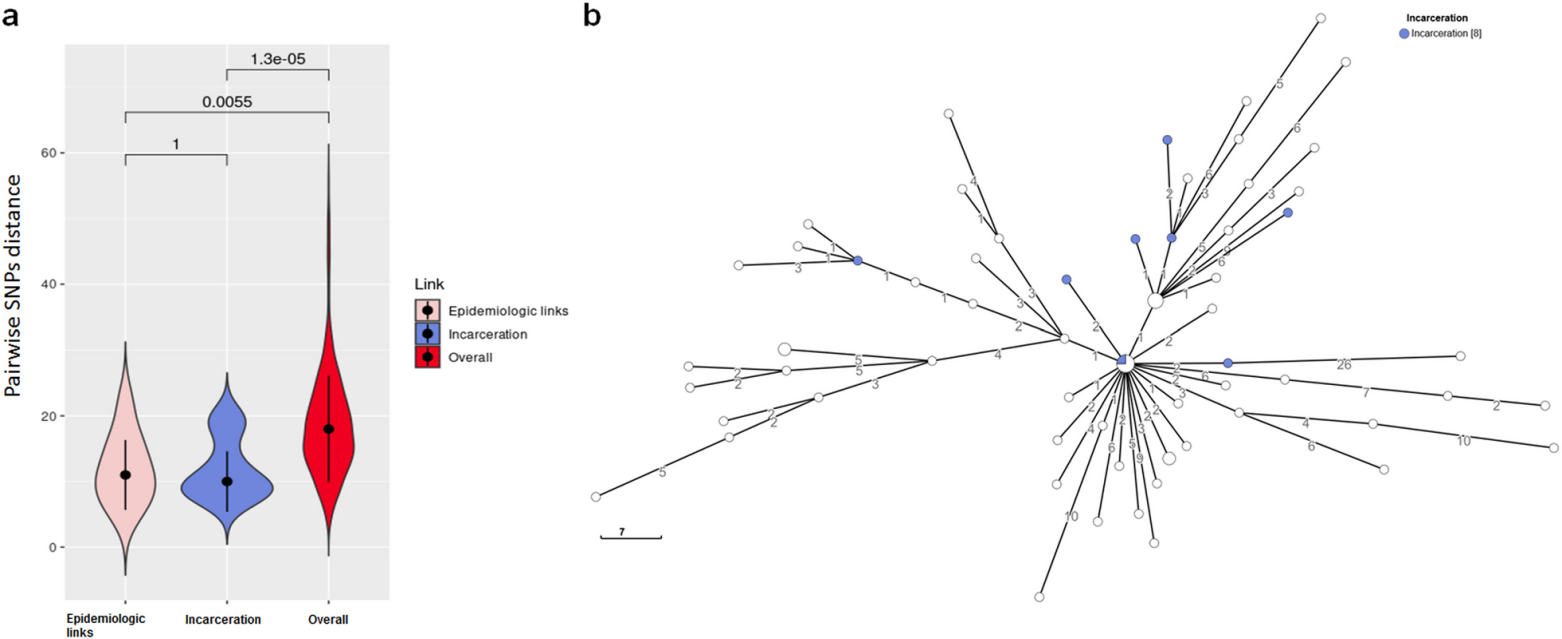
Genomic epidemiological analysis. **a** Violin plots showing the pairwise SNP distance based on 969 polymorphic sites of strains within each epidemiologic link. Lines within the violin represent the 25th and 75th percentile, black horizontal dot the median. **b** cgMLST showing the dispersion of patient strains with incarceration history

Isolates from patients with a confirmed epidemiological link (same family, close friends, neighbours, prison inmates, etc.) were genetically closely related, as indicated by the smaller median pairwise SNPs distance (median 11 SNPs, IQR: 8–16) relative to the overall outbreak cases (median 18 SNPs, IQR: 13–23) (*p* = 0.0055). Among the epidemiologically confirmed cases, isolates from patients with a history of incarceration were genetically the most closely related (median 10 SNPs, IQR: 9–15) as compared to the overall MDR TB dataset (median 18, IQR: 13–23) (*p* < 0.001) (Fig. 2A). Strikingly, a more detailed scrutiny of the cgMLST-based MST, revealed the spill-over of MDR TB from prison into the general public (Fig. 2B). Cases with history of incarceration were found to be directly or indirectly linked to up to 22 new outbreak cases (32.35%) among the non-imprisoned population. The very first case linked to the MDR TB outbreak, identified in 1998 and which was incarcerated at that time, appears to be linked to up to 17 new cases (SNPs difference < 5), including 2 imprisoned individuals.

### Delineating the spatio-temporal spread of the local MDR outbreak

**Fig. 3:**
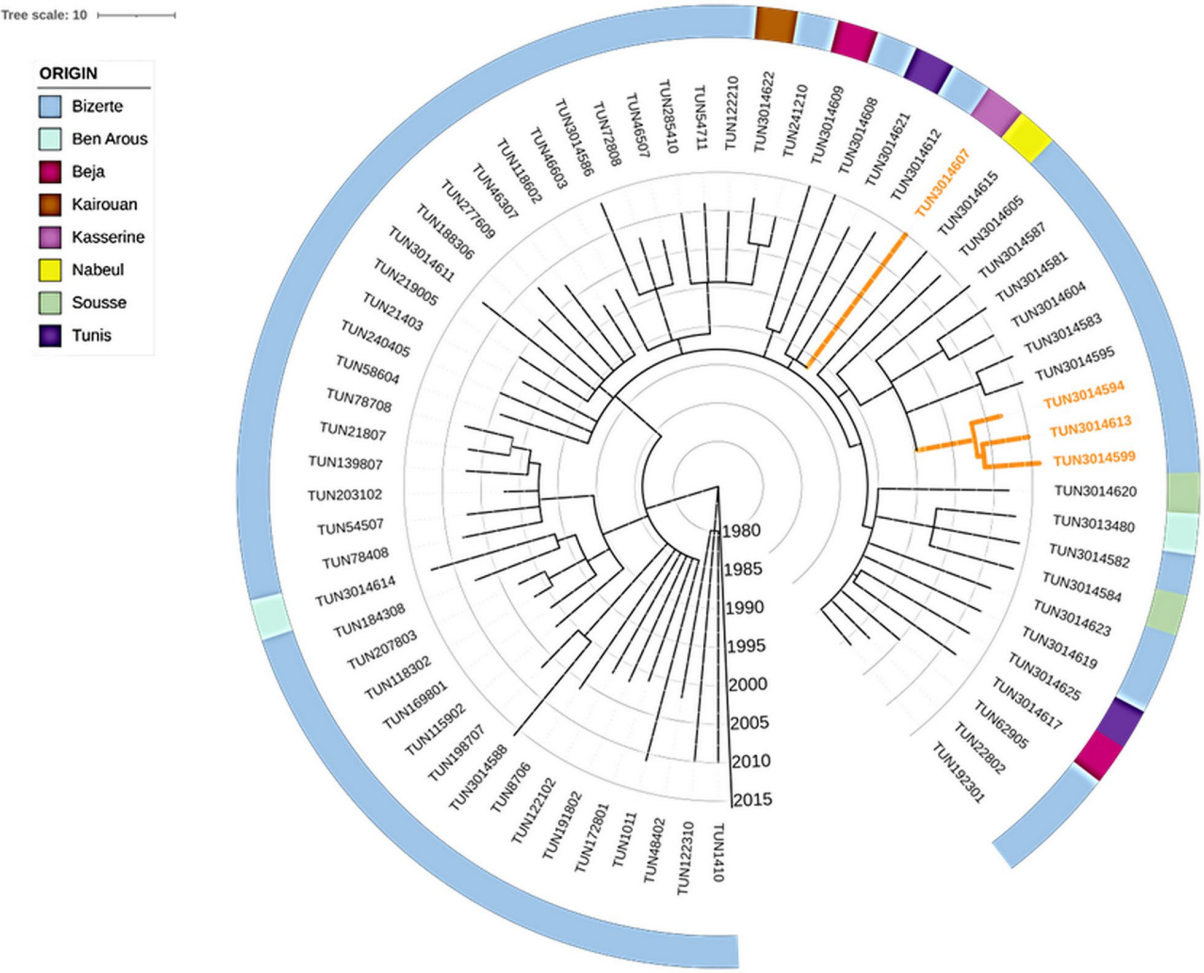
Time-scaled phylogenetic tree of MDR and XDR isolates based on WGS. External colour strips indicate the origin of clinical isolates. Orange branches correspond to XDR strains. Temporal predictions are obtained with LSD2 and the visualisation is performed with iTOL version 5

In order to gain more insights into the emergence of the Bizerte’s Haarlem MDR TB outbreak, we sought to employ the molecular clock method based on least-squares optimisation, LSD2. The mutation rate was estimated at 9.08e-08 (95%CI 5.68e-08; 1.29e-07) mutation per site per year. Phylogeographic and dispersal reconstruction of the outbreak ancestors, mapped its oldest ancestor to Bizerte, most likely around 1974 (95% CI 1951–1985), from where it spread to other regions by 1992 (95% CI 1980–1996) (Fig. 3 and Table 1). Next, we estimated the age of the most recent common ancestors (MRCAs) of samples carrying resistance mutations to second line drugs that were widespread within the outbreak. The *rrs* 1401A > G mutation is the only mutation found in pre-XDR (resistant to isoniazid, rifampin, and a second-line injectable (amikacin, capreomycin, and kanamycin [28]) and XDR patients, resulting in Kanamycin (KAN) resistance. The emergence of KAN resistance related to pre-XDR strains was estimated to have possibly evolved around 1998 (CI95% 1992–2003), while the MRCA of all XDR isolates dated back to 2008 (95% CI 2001–2012).

**Fig. 4:**
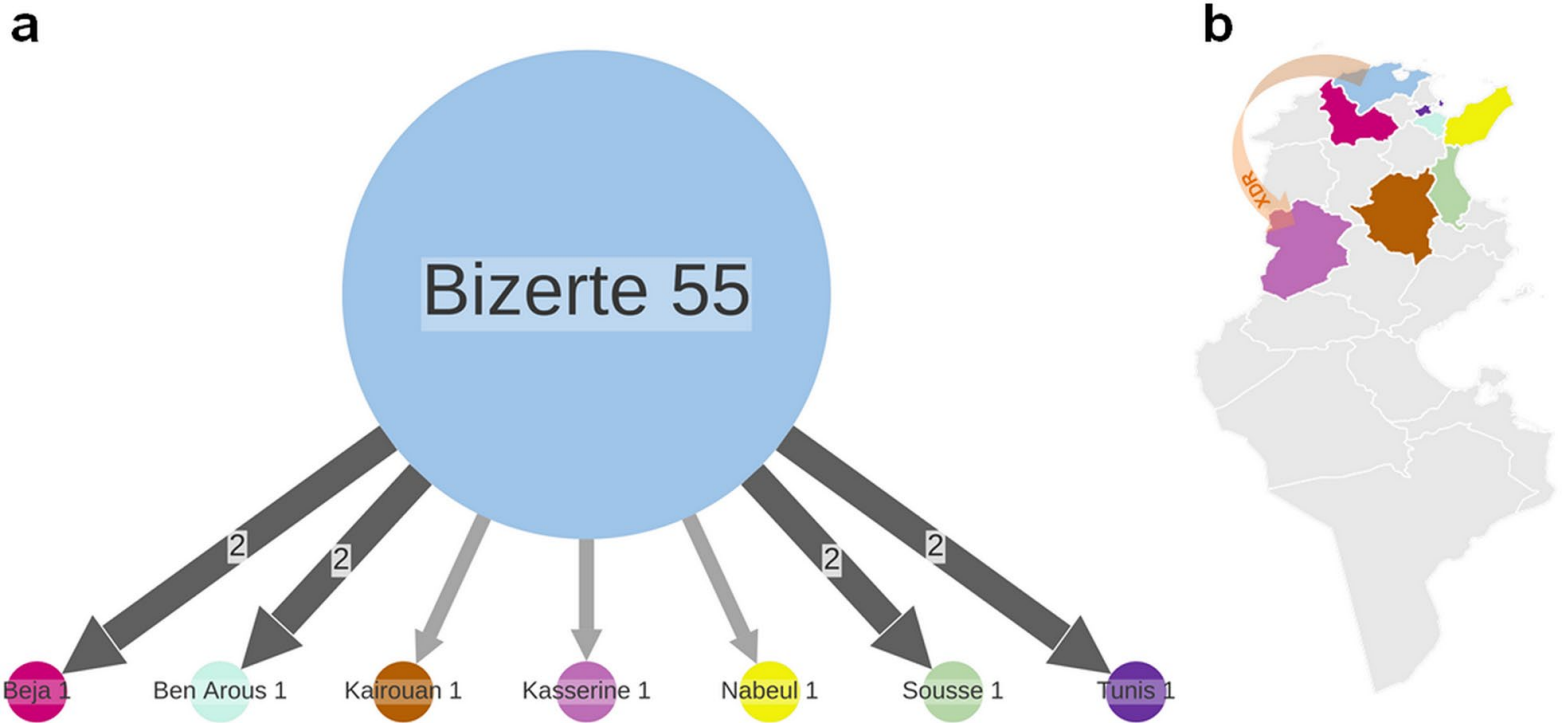
Dispersal of MDR and XDR epidemic strains. **a** Results of PASTML. The colour code for origin is the same as in Fig. 3. The size of the circles is proportional to the number of tips, and the size of the arrows is proportional to the number of times the pattern of migration was observed on the tree. The branches represent the inferred ancestral region with the largest posterior probability. **b** Map of Tunisia, the arrow shows the migration of XDR strain from the epidemic region to a Midwest City

Next, we performed a phylogeographic analysis using PASTML, which is based on different models and inference methods [29], and found that the region of Bizerte was predicted to be the geographic origin of the MRCA of the Haarlem MDR TB outbreak clone. PASTML identified migration events mostly from Bizerte toward other regions (Fig. 4A), which is marked by the export of the outbreak strain with an XDR phenotype to Kasserine, Midwest Tunisia (Fig. 4B).

## Discussion

WGS has been widely applied in international TB outbreak investigations [10, 30, 31]. It provides an added value to the timing and directionality of transmission analysis. This study illustrates the usefulness of WGS to delineate the early events leading to the emergence of an MDR TB outbreak, and allowed to have a good grasp of the way it has spread in the community. Using a 5-SNP cut-off in cgMLST analysis, we were able to pinpoint the critical role of prisons-related cases in the emergence of the outbreak, and unambiguously provide evidence for its tendency to spread in the whole country, far from the emergence area.

Here, we performed a genomic-based investigation of the largest MDR TB outbreak in Tunisia, which is caused by a L4.1.2.1/ Haarlem sublineage strain. A peculiar feature of this MDR TB outbreak is the fact that it has emerged and expanded, from the outset, in a context strictly negative for HIV infection, contrasting with the majority of MDR TB outbreaks described thus far [32, 33].

Phylogeographic analyses established the region of Bizerte as the main origin of this L4.1.2.1/Haarlem MDR TB outbreak, which is in line with the fact that the majority of cases have first been identified thereof. Dating analysis revealed that the most recent common ancestor for this MDR TB outbreak strain dates back to 1974 (95% CI 1951–1985), a finding that is consistent with the timing of the widespread introduction of rifampicin as part of anti-TB treatment regimens worldwide, including Tunisia [34]. This finding implies that it took almost 26 years before the outbreak became obvious in the early 2000s [5, 6]. During this period, one can suggest that the MDR outbreak strain could have been steadily circulating, most likely at a low rate. In this regard, it is worth mentioning that microepidemics in this particular region have already been noticed since the early nineties, as part of one of the first attempts to implement IS*6110*-based typing of *M. tuberculosis* [35]. Only in the early 2000s, was a sudden increase in the number of MDR TB cases noticed among TB patients presenting to the regional hospital of Menzel Bourguiba, which deserves the whole region of Bizerte. Indeed, up to 15 MDR TB cases were diagnosed in years 2001 and 2002, and the ongoing outbreak was confirmed by molecular typing [5, 6]. Genomic analyses indicated that the very first documented outbreak case, which was incarcerated a few years before the MDR TB outbreak became obvious, was very likely responsible of the 2001–2002 peak of the MDR TB outbreak, thus establishing a crucial role for incarceration in the MDR TB epidemic surge. Such a hypothesis goes in line with previous studies invoking the role of prisons in propagating TB epidemics in the general population [36, 37].

More specifically, a study conducted in Georgia, showed that prisons fuel the MDR TB epidemic by acting as ecological drivers of fitness-compensated strains endowed with high transmission potential [38], which is typically the case of the MDR TB of Bizerte. Indeed, the strain from the very first documented outbreak case harboured the *rpoB* V615M mutation, secondary to the rifampicin resistance-conferring mutation *rpoB* S531L. Previously, we have demonstrated the fitness compensatory role of *rpoB* V615M mutation and the concomitant increase in rifampicin resistance level that it induced (MIC > 160) [7]. Hence, it is tempting to speculate that incarceration could have conferred the very first case’s strain with increased transmissibility, thus marking the onset of the Bizerte’s MDR TB outbreak.

By 1998, a KAN-resistant *rrs* 1401A > G mutant has emerged [5], laying the ground for the shift toward an XDR phenotype. This date reflects the starting of the administration of the aminoglycoside KAN, most likely to the earliest documented outbreak case, since it has been realized that he remained smear-positive after several months of first-line chemotherapy. Obviously, the patient was on a functional KAN monotherapy, whose immediate consequence was acquisition of resistance to this aminoglycoside.

A few MDR TB outbreaks due to the L4.1.2.1/ Haarlem genotype has previously been described, the most notorious case being the “M” strain in Argentina. In this well-documented case, the spread of this MDR TB outbreak first involved HIV-positive hospitalized patients, and then extended to the general public [9]. The MDR TB outbreak due to strain “M” was detected around 15 years after the causing strain had accumulated extensive drug resistance profiles. In the case of the MDR TB outbreak from Bizerte, the causing strain has been circulating for a relatively much longer period of 26 years before the outbreak was detected. The most obvious explanation stems in the fact that in Argentina, HIV co-infection could have expedited the onset of the outbreak. Strikingly, the strains of these two MDR TB outbreaks appear to have emerged at nearly the same time (1973 for the M strain and 1974 for the one from Bizerte, respectively), yet they proved genetically distantly related, since their profiles based on IS*6110*, MIRU-VNTR, and spoligotyping diverged significantly (data not shown). Hence, it is very unlikely that the two outbreaks are actually interrelated.

From a public health/surveillance perspective, this WGS-based study provides a significant complement to previous investigations, refining our understanding of the potential genetic and environmental risk factors that contributed to the surge and spread of the described MDR TB outbreak. The health authorities are now aware of the dispersal of such a highly transmissible MDR *M. tuberculosis* strain across remote communities and are thus urged to raise the bar of surveillance throughout the whole country. The use of WGS coupled to dating analyses further indicated that strains with multiple resistance profiles could circulate for a few decades before causing an outbreak. This finding emphasizes the importance of focusing the monitoring of MDR TB upon cases living in crowded conditions, which likely expedite evolution towards an epidemic phenotype.

## Conclusions

In sum, the use of WGS data offered a better insight into the circumstances leading to the onset and spread of a major MDR TB outbreak in a context that is strictly negative for HIV infection. We showed that the outbreak took place almost three decades after the causing strain had accumulated multiple drug resistance profiles. To our knowledge, our study provides the first indication of the time interval required for the onset of an outbreak in the absence of HIV co-infection, the major amplifier of MDR TB outbreaks. Our findings lend further support to the prominent role of prisons as ecological incubator for the emergence of fitness-compensated MDR TB strains with enhanced transmission potential. Finally, the worrying shift to an XDR phenotype of such an epidemic clone prompts an urgent need to undertake drastic control measures. With the introduction of newer drugs and taking into account the new definition of XDR TB, it is worth to scrutinize eventual bedaquiline and linezolid resistance in this MDR TB clone.

### Supplementary Information

**Additional file 1: **New WGS strains collection.

**Additional file 2: **Publicaly WGS collection.

**Additional file 3: **3257 of the 4018 genes of H37Rv (NC_000962.3) were used for cgMLST.

## Data Availability

3257 of the 4018 genes of H37Rv (NC_000962.3) used for cgMLST in this study are provided in Additional file 2: Table S2. The WGS of the 39 MDR Tunisian isolates have been deposited in European Nucleotide Archive under project number PRJEB63074.

## Abbreviations

cgMLST: Core genome Multilocus Sequence Typing
HIV: Human immunodeficiency virus
MDR: MultiDrug-resistant
MST: Minimum Spanning Trees
*M. tuberculosis*: *Mycobacterium tuberculosis*
SNP: Single nucleotide polymorphism
TB: Tuberculosis
WGS: Whole Genome Sequencing
XDR: Extensively drug-resistant TB
